# Aloe Extracellular Vesicles as Carriers of Photoinducible Metabolites Exhibiting Cellular Phototoxicity

**DOI:** 10.3390/cells13221845

**Published:** 2024-11-07

**Authors:** Eleonora Calzoni, Agnese Bertoldi, Alessio Cesaretti, Husam B. R. Alabed, Giada Cerrotti, Roberto Maria Pellegrino, Sandra Buratta, Lorena Urbanelli, Carla Emiliani

**Affiliations:** 1 Department of Chemistry, Biology and Biotechnology, University of Perugia, Via del Giochetto, 06123 Perugia, Italy; eleonora.calzoni@unipg.it (E.C.); agnese.bertoldi@dottorandi.unipg.it (A.B.); husambr.alabed@dottorandi.unipg.it (H.B.R.A.); giada.cerrotti@dottorandi.unipg.it (G.C.); roberto.pellegrino@unipg.it (R.M.P.); sandra.buratta@unipg.it (S.B.); lorena.urbanelli@unipg.it (L.U.); carla.emiliani@unipg.it (C.E.); 2 Centro di Eccellenza Materiali Innovativi Nanostrutturati (CEMIN), University of Perugia, Via del Giochetto, 06123 Perugia, Italy

**Keywords:** plant-derived extracellular vesicles, aloe, photodynamic therapy, anthraquinones, oxidative stress, metabolomics

## Abstract

The growing interest in plant-origin active molecules with medicinal properties has led to a revaluation of plants in the pharmaceutical field. Plant-derived extracellular vesicles (PDEVs) have emerged as promising candidates for next-generation drug delivery systems due to their ability to concentrate and deliver a plethora of bioactive molecules. These bilayer membranous vesicles, whose diameter ranges from 30 to 1000 nm, are released by different cell types and play a crucial role in cross-kingdom communication between plants and humans. Notably, PDEVs have demonstrated efficacy in treating various diseases, including cancer, alcoholic liver disease, and inflammatory bowel disease. However, further research on plant vesicles is necessary to fully understand their traits and purposes. This study investigates the phototoxic effects of extracellular vesicles (EVs) from *Aloe arborescens*, *Aloe barbadensis*, and *Aloe chinensis* on the human melanoma cell line SK-MEL-5, focusing on their anthraquinone content, recognized as natural photosensitizers. The phototoxic impact of Aloe EVs is associated with ROS production, leading to significant oxidative stress in melanoma cells, as validated by a metabolome analysis. These findings suggest that EVs from *Aloe arborescens*, *Aloe barbadensis*, and *Aloe chinensis* hold promise as potential photosensitizers, thus highlighting their potential for future application in photodynamic cancer therapy and providing valuable insights into the possible utilization of PDEVs for therapeutic purposes.

## 1. Introduction

Nowadays, there is an ever-growing interest in plant-origin active molecules endowed with medicinal properties. Indeed, a large number of plant-derived drugs are currently used in the medical field; for example, more than 25% of antineoplastic drugs have natural origins [1,2,3]. In particular, the competition between natural and synthetic drugs in the pharmaceutical field has led to a revaluation and rediscovery of plants, increasing the scientific/commercial interest of pharmaceutical companies in natural extracts from plant matrices. The study of secondary metabolites extractable from plants has proven to be one of the most important and promising fields of modern medicine both for new extraction techniques and for the possibilities of using new substances. In this contest, plant-derived extracellular vesicles (PDEVs) represent ideal candidates as a next-generation drug delivery system in different types of pathologies as they are capable of concentrating and transporting different types of bioactive molecules [4,5,6]. A wide variety of chemicals can be found in plant extracts, although some of them may be potentially harmful. The administration of bioactive ingredients through EVs instead offers a more controlled and refined approach, potentially reducing the risk of toxicity. EVs are bilayer membranous vesicles with a range of sizes going from 30 to 1000 nm, which are released by different types of cells [7]. Recently, PDEVs have gained attention as an ‘animal-free ingredient’ in a variety of industries, including cosmetics and pharmaceuticals. In addition to proteins, lipids, mRNAs, and microRNAs (miRNAs), PDEVs can also transport other metabolites of the generating cell and play an important role in the cross-talk between vegetable and human kingdoms. PDEVs can protect their labile cargo from degradation and permit their uptake through endocytosis by tissues [8]. The use of EVs from plant species is also extremely interesting due to the potential access to exceptionally complex molecular structures that would be difficult to synthesize. Several studies have demonstrated the effectiveness of PDEVs in the treatment of cancer, alcoholic liver disease, and inflammatory bowel disease [9,10,11,12,13,14]. However, as the features and purposes of plant EVs have not been entirely understood, further thorough research on plant EVs needs to be carried out. Despite evidence of the antiproliferative and anticancer effects of many plant extracts, it is still unclear if PDEVs could have the same properties as well. Many studies also suggest that natural compounds show photosensitizing features and can be employed as surrogates for conventional photosensitizers (PSs) used in the photodynamic therapy (PDT) of cancer [15,16,17,18].

PDT features the administration of a photosensitizer molecule that accumulates exclusively in target cells followed by the local irradiation of the tumor. By resorting to photosensitizers that are specifically delivered in tumor masses and targeted light irradiation, PDT has more selectivity against malignant cells than conventional methods of treatment [19]. In PDT, light energy is transferred to molecular oxygen upon irradiation. It results in the production of Reactive Oxygen Species (ROS) like singlet oxygen (^1^O_2_), superoxide radical (O^2−^), hydroxyl radical (HO•), and hydrogen peroxide (H_2_O_2_). These cytotoxic substances give rise to a cascade of biochemical processes that result in tissue destruction or apoptosis [16,18,20].

The photoactive compounds present in natural species have proven to be less toxic than synthetic agents and have shown fewer side effects, thus having advantages in the therapeutic approach [16,21]. One of the most important properties of natural photoactive compounds is represented by the possibility of switching on their toxicity under light irradiation, while they possess an inherent non-toxic nature in the absence of light. These photoactive extracts and phytocompounds thus become good candidates as photosensitizers for PDT [22]. Another important characteristic that makes plant photoactive compounds suitable for therapeutic use is their biologically compatible absorption spectrum, which is usually located in the visible and near-infrared region [23]. Photoactivated compounds can produce radicals and, in the presence of oxygen, ROS, which could lead to apoptotic processes in cancer cells [16,18,23].

The most important plant-based compounds showing photoactivity are furanocoumarins, polyacetylenes, thiophenes, curcumins, alkaloids, and anthraquinones [22,24,25]. Anthraquinones are a noteworthy class of photosensitizer molecules as they lead to the formation of singlet oxygen anion radicals and/or superoxide. For more than 4000 years, these substances have been recognized and used in conventional medicine [16,18,23,26]. The photoactive nature of anthraquinones and their ability to inhibit the proliferation of cancer cells were demonstrated with anthraquinone-rich plant extracts under visible radiation at 380–480 nm that promoted the antiproliferative effect on MCF-7 breast cancer cells [27].

Aloin A, aloin B, and aloe-emodin are the main anthraquinones found in the plant world and are particularly present in different Aloe species. The Aloe plant is one of the most used by humans since ancient times, finding widespread use in pharmaceutical, nutraceutical, and cosmetic products. In fact, Aloe is also rich in pharmacologically active compounds that exhibit various biological properties such as antiviral, fungicidal, antibacterial, anti-inflammatory, antimicrobial, laxative, and anticancer activity [28,29]. It has been demonstrated that aloin, also known as barbaloin, and aloe-emodin are photoinducible anthraquinone species and therefore potentially usable as photosensitizers. In particular, it has been seen that aloin subjected to UVA irradiation induces the formation of radical species and lipid peroxidation, resulting in strong oxidative stress [30]. Following irradiation, aloe-emodin also induced apoptosis in basal and squamous carcinoma cells and inhibited the process of metastasis in breast cancer [31,32]. Until today, photoinducible effects of anthraquinones have been investigated on leaf extracts, but the same properties on EVs have never been explored; therefore, it is extremely interesting to evaluate the possible phototoxic effect of the anthraquinones transported by EVs, since, as widely demonstrated, PDEVs are able to make the transported molecules more bioavailable, being protected by the phospholipid layer, which prevents them from degradation. Furthermore, EVs of a plant origin have a low immunological risk, which makes them excellent candidates for the treatment of various pathologies [33].

Since the antiproliferative and antitumor properties of the extracts obtained from different Aloe species are already known, in this work, the possible phototoxic effect of EVs from *Aloe arborescens*, *Aloe barbadensis*, and *Aloe chinensis* was investigated using the human melanoma cell line SK-MEL-5 as a target. EVs purified from the three Aloe species exhibited a noteworthy phototoxic effect on cancer cells, and this result was corroborated by the alterations observed in the metabolome of the treated and irradiated cells.

Therefore, the potential use of EVs isolated by different Aloe species as natural photosensitizers was evaluated, in order to contribute to future potential applications in the PTD of cancer.

## 2. Materials and Methods

### 2.1. Materials

*Aloe arborescens Mill*., *Aloe barbadensis Mill*., and *Aloe barbadensis* var. *chinensis Haw*. (*Aloe chinensis*) were purchased from a local nursery and cultivated under commercial organic grown conditions. 2-(N-Morpholino) Ethanesulfonic Acid (MES), Calcium chloride (CaCl_2_), Sodium Chloride (NaCl), methanol, and ethanol were purchased from Sigma Aldrich (Saint Louis, MO, USA). Aloe-emodin and Aloin Q-TOF LC/MS Internal standards for anthraquinone determination were purchased from Sigma Aldrich. 3-(4,5-dimethylthiazol-2-yl)-2,5-diphenyltetrazolium bromide (MTT), 2′,7′-Dichlorodihydrofluorescein diacetate, Phosphate-Buffered Saline (PBS), Dimethyl Sulfoxide (DMSO), and glutaraldehyde (25%) were purchased from Sigma-Aldrich. Dulbecco’s modified Eagle’s medium (DMEM), fetal bovine serum (FBS), trypsin, and penicillin/streptomycin were purchased from Euroclone (Pero, Italy). All the other chemicals were of an analytical grade and were obtained from Sigma-Aldrich unless otherwise indicated. SK-MEL-5 human melanoma cells were purchased from ATCC (Manassas, VA, USA).

### 2.2. Aloe EV Isolation and Aloe Crude Extracts

**Figure 1:**
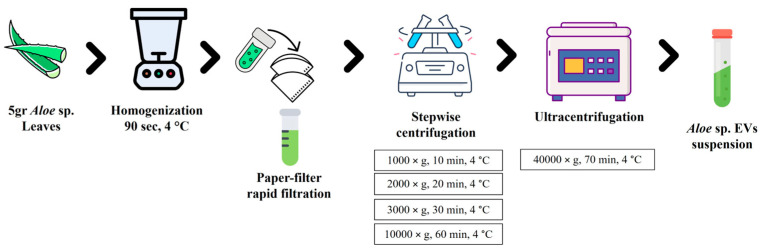
Extracellular vesicles (EVs) from *Aloe arborescens*, *Aloe barbadensis*, and *Aloe chinensis* isolation process.

For the isolation of EVs from *Aloe arborescens*, *Aloe barbadensis*, and *Aloe chinensis*, the method of Kim et al. was used, with few modifications [9]. Leaves from *Aloe arborescens*, *Aloe barbadensis*, and *Aloe chinensis* were washed with deionized water and then 5 g of each sample was homogenized with 10 mL of a VIB buffer (20 mM MES, 2 mM CaCl_2_, 0.1 M NaCl, pH 6.0) using ULTRA-TURRAX T25 (IKA^®^-Werke GmbH & Co. KG, Staufen, Germany) for 90 s at 4 °C. To remove contaminants such as cell organelles, the extracellular matrix, and fibers, each homogenate was purified by serial centrifugations. A stepwise centrifugation (Figure 1) of the mixture was performed at 1000× *g* for 10 min at 4 °C, 2000× *g* for 20 min at 4 °C, 3000× *g* for 30 min at 4 °C, and 10,000× *g* for 60 min at 4 °C (followed by a filtration step with a 0.45 μm filter), with the collection of the supernatant at each step. The supernatant was then ultracentrifuged at 40,000× *g* for 70 min at 4 °C. After the first ultracentrifugation, the pellet was resuspended in 9 mL of 1× PBS to remove contaminants and subjected to additional ultracentrifugation at 40,000× *g* for 70 min at 4 °C. The final pellet was then resuspended in 200 µL of PBS with 10% of DMSO for biological assays and with 100 µL of methanol (100%) for the Q-TOF LC/MS analysis. The EV pellets resuspended in PBS with 10% DMSO were used for the determination of the protein content through the Bradford assay [34].

For the preparation of *Aloe arborescens*, *Aloe barbadensis*, and *Aloe chinensis* crude extracts, 2 g of each sample was homogenized with 10 mL of ethanol (80%) and left overnight at 4 °C under stirring. Each sample was then centrifuged at 15,000× *g* for 10 min at 4 °C and the supernatant was left to concentrate until completely dry. The pellet was subsequently resuspended in 500 µL of PBS containing 10% DMSO for biological assays and in 500 µL of pure methanol for the Q-TOF LC/MS analysis.

### 2.3. Nanoparticle Tracking (NTA) Analysis

Particle concentration and size distribution were measured using a Malvern Panalytical NanoSight NS300 Nanoparticle Tracking Analysis (NTA) (NanoSight Model NS300, Malvern Instruments, NanoSight Ltd., Salisbury, MD, USA). For the NTA system’s working concentration range, EVs from different Aloe species were resuspended and diluted in filtered PBS, and five measurements were made for each sample. The analysis of EVs from two independent experiments was carried out.

### 2.4. Scanning Electron Microscopy (SEM) Analysis

In total, 3 µg of *Aloe arborescens*, *Aloe barbadensis*, and *Aloe chinensis* EVs were fixed in 2.5% glutaraldehyde for 15 min at room temperature, diluted with 15 mL of H_2_O_d_, and washed twice using Vivaspin concentrating devices with a cut-off of 300 kDa. The tubes were centrifuged at 3000× *g* for 2 min, and the samples were recovered and diluted in H_2_Od, then sedimented on glass coverslips and left to dry at room temperature. SEM images were obtained, as already described elsewhere [35], by using a field emission LEO 1525 electron scanning microscope (Zeiss, Thornwood, NY, USA) equipped with a Gemini column after chromium metallization through a high-resolution sputter Q150T ES-Quorum apparatus (24 s sputter at a current of 20 mA). Chromium thickness was ~10 nm.

### 2.5. Anthraquinone Quantification by Targeted Q-TOF LC/MS Analysis

The extraction of anthraquinones from the crude extracts and EVs of *Aloe arborescens*, *Aloe barbadensis*, and *Aloe chinensis* (prepared as described in Section 2.2) was performed by the addition of proper volumes of methanol containing the internal standards of aloin and aloe-emodin to each sample. Specifically, 500 µL and 100 µL of methanol were added to the crude extract and EVs, respectively. After the addition of methanol, the samples were vortexed and centrifuged at 5000× *g* for 10 min. The supernatant was collected and transferred to glass vials for injection.

For targeted analyses of these species, an Agilent Analyzer (Santa Clara, CA, USA) consisting of an Agilent 1260 Infinity II liquid chromatograph coupled with an Agilent 6530 Accurate-Mass Q-TOF (quadrupole-time-of-flight) Analyzer Santa Clara, CA, USA) and an Agilent JetStream source was used. The chromatographic column used was a Waters XBridge BEH Amide (HILIC) (Milford, MA, USA) column (150 mm, 2.1 mm, and 2.5 µm) and was maintained at 25 °C with a flow rate of a mobile phase of 0.35 mL/min. The mobile phase consisted of a mixture of water (Solvent A) and acetonitrile (Solvent B), both with 0.2% formic acid. The chromatographic separation conditions were conducted by following the indications reported in PMID: 38612731 and PMID: 34957181 [36,37]. The source was operated in both polarities as follows: ion spray at 3500 V; gas temperature and sheath gas temperature were set at 250 and 300 °C, respectively; the nebulizer (N_2_) at 35 psi; and sheath gas flow at 12 L/min. Data-dependent acquisition was used in the mass range of 40–1700 *m*/*z* for both MS and MS/MS with a collision energy of 30 V.

The acquired data were processed (peak detection and annotation) using the Qualitative Analysis MassHunter software provided by Agilent. A multi-level external calibration was performed using the following standard concentrations: 1, 5, 10, 20, 50, and 100 µg/mL. Three replicates were performed for each standard. The calibration curves yielded an R² of 0.9974 for aloin A and 0.9975 for aloe-emodin, respectively.

### 2.6. MTT Cytotoxicity and Phototoxicity Assay

The MTT assay was used to study the effect of the EVs from *Aloe arborescens*, *Aloe barbadensis*, and *Aloe chinensis* and that of the crude extracts on cell proliferation with and without light irradiation. In particular, 2 × 10^3^ SK-MEL-5 cells were seeded in Falcon 96-well clear flat-bottom microplates (Becton, Dickinson and Company, Franklin Lakes, NJ, USA), in 200 μL of a culture medium (DMEM). The following day, the medium was replaced with 200 μL of a fresh medium containing different dilutions of the EVs derived from *Aloe arborescens*, *Aloe barbadensis*, and *Aloe chinensis*, or crude extracts. Each dilution was analyzed in quadruplicate; a quadruplet was kept as the control (200 μL of DMEM) and other quadruplets were used to take into account the contribution of PBS + DMSO (vehicle controls) at the same concentrations of the different dilutions of EVs or crude extracts. The cells were either kept in the dark or photoexposed for 50 min in an LED chamber (λ_exc_ = 390−400 nm), producing a power of about 1.7 mW/cm^2^. After 24 h of incubation in a humidified atmosphere with 5% CO_2_ at 37 °C, the MTT test was performed as widely described elsewhere [38,39]. Cell viability was expressed as the optical density percentage in treated cells compared with vehicle controls, assuming the absorbance of controls was 100% (absorbance of treated wells/absorbance of control wells × 100).

### 2.7. Intracellular ROS Production Assay

Intracellular ROS levels were measured using the H_2_DCFHDA method, based on the detection of the fluorescence of DCF, which is produced in the presence of ROS, as previously outlined in other studies [38]. Briefly, 10 × 10^3^ SK-MEL-5 cells were seeded in each well of a Corning 96-well black round-bottom polystyrene microplate (Corning Incorporated, Corning, NY, USA), adding 200 μL of a culture medium (DMEM). The following day, the same experiment as the phototoxicity assay described above was performed. After 30 min from the photoexposition, the H_2_DCFHDA assay was carried out [38], finally revealing the fluorescence of oxidized DCF. Data (expressed as the percentage of DCF fluorescence intensity relative to vehicle controls) were normalized to cell viability evaluated by the MTT assay performed on another Falcon 96-well clear flat-bottom microplate (Becton, Dickinson and Company, Franklin Lakes, NJ, USA) seeded with cells simultaneously treated and photoexposed under the same experimental conditions. All measurements were performed in quadruplicate in three independent experiments.

### 2.8. Immunoblot Analysis

In order to evaluate the effect of the EVs from *Aloe arborescens*, *Aloe barbadensis*, and *Aloe chinensis* after light irradiation on some characteristic protein markers, SK-MEL-5 cells were seeded in Falcon 12-well clear flat-bottom microplates (Becton, Dickinson and Company, Franklin Lakes, NJ, USA) (1 × 10^5^ cells/well), in 1000 μL of a culture medium (DMEM). The following day, the medium was replaced with 1000 μL of a fresh medium containing the EVs derived from *Aloe arborescens*, *Aloe barbadensis*, and *Aloe chinensis*. The cells were photoexposed for 50 min in the LED chamber as described above. Following 24 h from the exposure, 10 μg of each cell lysate was subjected to 10% SDS-PAGE (Mini-PROTEAN^®^ 3 Cell, Bio-Rad, Hercules, CA, USA) under reducing conditions according to Laemmli’s method [40] and then the proteins were transferred to a polyvinylidene difluoride (PVDF) membrane as previously described elsewhere [41]. The PVDF membrane was firstly incubated overnight with a voltage-dependent anion channel (VDAC), Cytochrome c Oxidase (COX IV), and finally with β-actin primary antibodies (Santa Cruz Biotechnology Co., Dallas, TX, USA) at 4 °C for 12 h. Protein measurements were performed using the ECL (Enhanced Chemiluminescence) detection system. A densitometry analysis on the immunoblot images was performed using Image J Software.

### 2.9. Q-TOF LC/MS Metabolomics Untargeted Analysis

To analyze the impact of the treatment on the cell metabolome, an untargeted LC/MS QTOF metabolomic analysis was performed on SK-MEL-5 cells after 24 h from the treatment with three types of EVs with and without light irradiation for 50 min in the LED chamber, using the same Q-TOF LC/MS instrument mentioned before. Spectrometric data were acquired in the 40–1700 *m*/*z* range both in negative and positive polarity in the full scan mode. For MS/MS data, pooled samples were analyzed at least five times in the iterative data-dependent acquisition mode.

Raw data were processed using the MS-DIAL software (4.48) to perform peak picking, alignment, and peak integration. The MS signal threshold was set at 1000 counts. In the end, a data matrix was obtained, reporting the accurate mass and area of each peak revealed in each sample analyzed [29]. The putative annotation of metabolites and the prediction of metabolic pathways was performed using the mummichog algorithm, implemented in the ‘MS Peaks to Pathways’ module of the Metaboanalyst 5.0 web platform. ANOVA and a Functional Meta-Analysis were also performed with MetaboAnalyst. For a statistical analysis, samples were normalized by the median, followed by Pareto scaling.

## 3. Results and Discussion

### 3.1. Aloe EV Morphological Characterization

**Figure 2:**
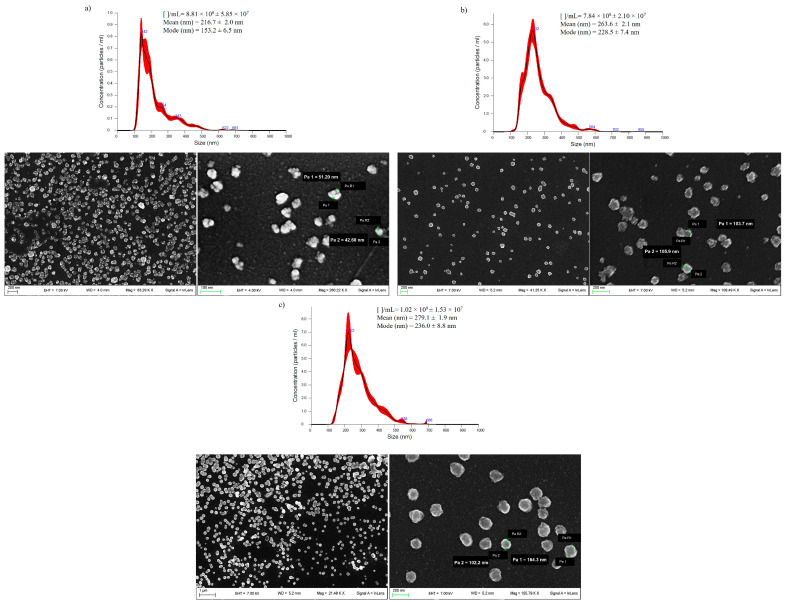
NTA analysis of EVs from *Aloe arborescens* (**a**), *Aloe barbadensis* (**b**), and *Aloe chinensis* (**c**), and respective SEM images. Data are expressed as mean ± SD (*n* = 3).

Aloe EVs were characterized by morphology and size distribution through scanning electron microscopy (SEM) and a nanoparticle tracking analysis (NTA), respectively (Figure 2). The average size and size distribution of the *Aloe arborescens*, *Aloe barbadensis*, and *Aloe chinensis* EVs were defined using NTA, whereas morphologies were characterized using SEM. The NTA analysis showed that the EVs of the three Aloe species have an average size of around 200/300 nm with small differences due to the species of origin (216.7 ± 2.0 nm for *Aloe arborescens*, 263.6 ± 2.1 nm for *Aloe barbadensis*, and 279.1 ± 1.9 nm for *Aloe chinensis*) (Figure 2a). *Aloe arborescens* EVs show an average size smaller than *Aloe barbadensis* and *Aloe chinensis* EVs and these data are also supported by the different mode values obtained (about 153 ± 6.5 nm for A. arborescens and about 230 ± 8.0 nm for A. barbadensis and *A. chinensis*). The total number of EVs isolated from *Aloe arborescens*, *Aloe barbadensis*, and *Aloe chinensis* leaves is ~1.8 × 10^8^ particles/g, 1.6 × 10^8^ particles/g, and 2.0 × 10^8^ particles/g, respectively. Moreover, SEM images revealed that EVs from *A. arborescens*, *A. barbadensis*, and *A. chinensis* exhibit a slightly lumpy spherical shape.

These results demonstrated that the reported isolation method allows for obtaining EVs characterized by an imperfectly rounded morphology and a size distribution comparable to those of vesicles released by other plant cells. The imperfect shape of EVs has already been found in other PDEVs, such as those obtained from Arabidopsis thaliana and Ginger, and is attributable to the presence of proteins associated with the phospholipid bilayer of the membrane [42,43,44]. Indeed, the EVs of all the species analyzed showed a high protein content, ~6.1 µg/g for *Aloe Arborescens*, ~2.7 µg/g for *Aloe barbadensis*, and ~11.0 µg/g for *Aloe chinensis*, as revealed by the Bradford assay. In addition, the NTA analysis revealed that the average size distribution of all three types of Aloe EVs is in line with data present in the literature (200 nm) as highlighted for the EVs obtained from Sunflower, Tobacco, Salvia dominica, or different species of Cannabis, confirming the success of the isolation method [43,45,46,47]. It is important to note that the size of EVs can be influenced by the isolation techniques used, including the centrifugal force applied. For example, studies that employ ultracentrifugation at higher forces (e.g., 100,000× *g*) tend to isolate smaller vesicles [9]. In contrast, in this study, EVs were isolated at 40,000× *g*, which can lead to the recovery of slightly larger vesicles. In our case, the size distribution observed is consistent with the applied ultracentrifugation conditions and aligns with previous reports on EVs from other plant species [48,49].

### 3.2. Q-TOF LC/MS Targeted Analysis for Anthraquinone Quantification

In order to evaluate the possible phototoxic effect of EVs from the selected Aloe species, a targeted analysis was first conducted using Q-TOF LC/MS to search for the main anthraquinone species to which a photosensitizing effect can be attributed. In this regard, the concentration of aloin A, aloin B, and aloe-emodin in the isolated EVs was evaluated, comparing their content with that of the respective plant extracts, to analyze whether the vesicles could respect the anthraquinone profile of the plant of origin.

**Figure 3:**
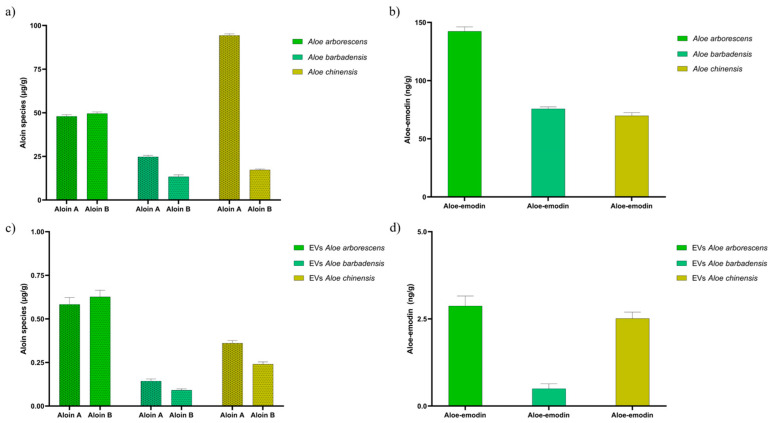
Quantification of aloin A, aloin B, and aloe-emodin through Q-TOF LC/MS analysis in *Aloe arborescens*, *Aloe barbadensis*, and *Aloe chinensis* plant extracts (**a**,**b**) and respective EVs (**c**,**d**). Data are reported as µg of aloin species or ng of aloe-emodin per g of fresh leaves’ material and expressed as mean ± SD (*n* = 5).

As reported in Figure 3, the concentration of the analyzed metabolites changes between the three Aloe species in both the crude extracts (Figure 3a,b) and the EVs (Figure 3c,d). In general, the trend of aloin A, aloin B, and aloe-emodin content found in the EVs reflects that of the plant extract, with concentrations of the order of µg/g for aloin A and B, and ng/g for aloe-emodin. However, the concentrations of these metabolites in the EVs are approximately one hundred times lower than in the crude extracts. In regard to *Aloe arborescens*, a similar content of aloin A and aloin B was detected both in the extract (~47.9 and ~49.6 µg/g) and in the corresponding EVs (~0.60 and ~0.62 µg/g), while a lower content of aloin B was found for *Aloe barbadensis* and even more markedly for *Aloe chinensis*. The latter showed the highest content of aloin A relative to the two other species in the plant extract (~94.3 µg/g), but a less marked difference was detected for the corresponding EVs. As for aloe-emodin, a concentration trend very similar to that of the respective plant extracts was detected in EVs with the exception of those isolated from *Aloe barbadensis*. The variation in metabolite concentration between extracts and EVs is most likely due to the selective packaging of bioactive compounds within the EVs, as well as inherent changes in the EV isolation process. While the metabolites are abundant in the crude extracts, their reduced quantities in EVs indicate that only a fraction of these molecules are integrated into the vesicles, underlining the selective character of EV cargo loading. The obtained results therefore reveal that *Aloe arborescens* and *Aloe chinensis* show higher content of aloin A, B, and aloe-emodin; however, it cannot be excluded that other potentially photoinducible anthraquinone species may be present in the plant species analyzed. Moreover, the presence of the same anthraquinone species detected in the corresponding plant extracts demonstrates how EVs serve as carriers for bioactive molecules, safeguarding them from degradation and facilitating their delivery to the intended target. In addition, although plant extracts contain a higher concentration of these molecules, they are nonetheless immersed in a plethora of other compounds, some of which may be potentially toxic and make them not always applicable in photodynamic therapy.

### 3.3. Phototoxicity Effect of Aloe EVs and ROS Production in SK-MEL-5 Skin Cancer Cells

**Figure 4:**
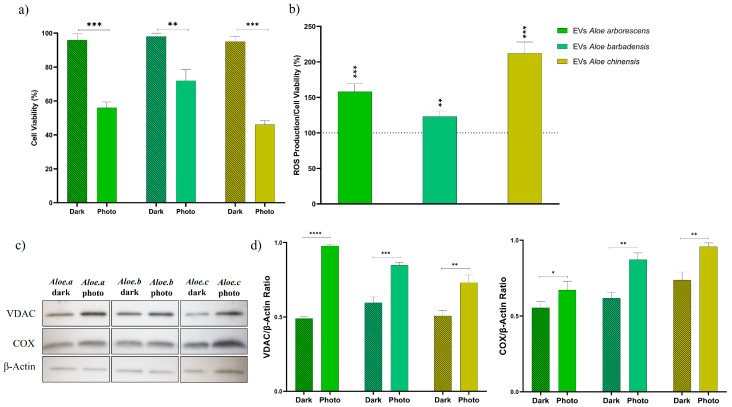
The cytotoxicity and phototoxicity (**a**) as well as ROS production (**b**) of Aloe EVs (1:50 dilution ratio) on SK-MEL-5 cells. Cytotoxicity (Dark) was evaluated on cells kept in the dark, while phototoxicity (Photo) and ROS production were assessed after 50 min of irradiation (corresponding to irradiation energy of 2.55 J/cm^2^, λ_exc_ = 390−400 nm). Cell viability is expressed as the mean of three independent experiments of four replicas each ±SD; 100% corresponds to control (untreated cells kept in the dark and photoexposed for Dark and Photo, respectively) mean values. * *p* < 0.05, ** *p* ˂ 0.01, *** *p* ˂ 0.001, **** *p* ˂ 0.0001. (**c**) Immunoblotting of SK-MEL-5 cells treated with *Aloe arborescens*, *Aloe barbadensis*, and *Aloe chinensis* EVs before and after UV irradiation. (**d**) Expression levels of VDAC and COX analyzed by Image J Software; β-actin was used as the internal control.

In order to fully exploit the intriguing potential of Aloe EVs in the PDT of tumor cells, experiments were conducted to test the cellular phototoxicity, together with their dark cytotoxicity, of *Aloe arborescens*, *Aloe barbadensis*, and *Aloe chinensis* EVs. Samples of EVs in 1:50 (Figure 4) and 1:100 (Figure S1a) dilution ratios were used to assess the toxicity of each species. The specific dilutions were selected based on preliminary evaluations, which indicated that these concentrations were appropriate for observing measurable phototoxic biological effects without causing any dark cytotoxicity. Similarly, toxicity was also evaluated for the crude extracts (1:100 dilution ratio) of three Aloe species obtained after the homogenization of plant tissues (Figure S1b).

While no cytotoxic effect was revealed for EV-treated cells kept in the dark (Figure 4a, Dark), all three EVs were found to be phototoxic towards tumor cells after subjecting them to 50 min of irradiation, corresponding to 2.55 J/cm^2^ irradiation energy (Figure 4a Photo and Figure S1a). At the same time, this photoexposition did not cause any appreciable viability reduction in untreated cells). In particular, the EVs of *Aloe arborescens* and *Aloe chinensis* gave a more evident phototoxic effect with viability after photoirradiation reduced to around 70 and 60% and 70 and 50%, for the two considered dilutions, respectively. This phototoxicity was also observed for the *Aloe barbadensis* EVs, although to a lesser extent (85% and 70% of viability). Moreover, by comparing the phototoxicity of plant extracts with that of EVs (Figure S1), it is evident that Aloe EVs, although containing a smaller quantity of photoactive anthraquinone molecules, are capable of more effectively delivering them into cells, thus resulting in an enhanced effect compared to that of the crude extracts. This can be likely explained by the ability of lipid-based EVs to efficiently interact and fuse with cell membranes, thus facilitating the delivery of their cargo into cells, as demonstrated in several studies on PDEVs [8]. On the other hand, biomolecules contained in plant extracts are often subject to degradation and struggle to permeate biological membranes without inducing cytotoxic effects.

Experiments aimed at evaluating intracellular levels of Reactive Oxygen Species (ROS) were carried out in order to elucidate the origin of the observed phototoxicity toward skin cancer cells (Figure 4b). The treatment with the three species of Aloe EVs (dilution of 1:50) and their successive irradiation were found to generate ROS in the investigated tumor cells well above the control levels. The production of intracellular ROS reflects the results of phototoxicity, where the increase in ROS concentration is directly proportional to the decline in viability following irradiation. In fact, a greater concentration of ROS was found in cells treated with *Aloe arborescens* EVs and particularly *Aloe chinensis* EVs, where a ROS concentration of approximately 150% and 200%, respectively, was reached. Also in this case, the production of ROS for cells treated with EVs from *Aloe barbadensis* was lower (120%), but still significantly increased compared to the control. The results demonstrated that in the presence of oxygen and light irradiation, natural anthraquinones, being likely excited to a long-lived triplet state populated through intersystem crossing (ISC) after photoexcitation, give energy transfer to molecular oxygen with the subsequent generation of ROS [38,50]. The results were also confirmed by the analysis of mitochondrial protein markers associated with oxidative stress and apoptosis (Figure 4c,d). In particular, the levels of the voltage-dependent anion channel (VDAC) protein were analyzed, which plays a key role in mitochondria-mediated apoptosis, acting in the release of apoptotic proteins located in the intermembrane space. The results highlighted that in cells treated with EVs from the three Aloe species, especially in the case of *Aloe arborescens* EVs, a significantly increased expression of VDAC is obtained following light irradiation, suggesting the triggering of an apoptotic process. Similarly, a significant increase in mitochondrial Cytochrome c Oxidase (COX IV) is also observed following treatment with EVs and irradiation as its dysfunction is associated with the increased production of ROS and cellular toxicity [51,52]. These outcomes about the cellular phototoxicity and light-induced ROS generation of Aloe EVs in melanoma cells suggest that they might be successfully exploited in the photodynamic therapy (PDT) of cancer to deliver natural photosensitizers in tumor cells.

### 3.4. Metabolomic Analysis of Cancer Cells

**Figure 5:**
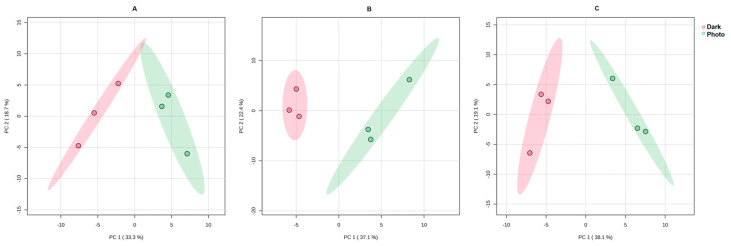
PCA score plots of SK-MEL-5 treated with *Aloe arborescens* (**A**), *Aloe barbadensis* (**B**), and *Aloe chinensis* (**C**) EVs kept in the dark (Dark, red) and after photoexposition (Photo, green).

The metabolomic analysis through a multivariate data analysis was conducted to address the effects of photoexposition after treatment with the three different Aloe EVs. In particular, it was possible to note 442 polar metabolites in the investigated EV-treated tumor cells. The matrices obtained were then processed and normalized in order to perform a Principal Component Analysis (PCA) (Figure 5), which shows a well-separate cluster between the control (Dark) and photoexposed (Photo) groups in the 95% confidence interval, indicating significant metabolic changes in photoexposed SK-MEL-5 cells as a result of the photoactivation of the anthraquinones delivered by Aloe EVs.

In particular, Figure 5 shows the result of PCA score plots, representing the distribution between the EV-treated cells kept in the dark (Dark, in red) and the photoexposed samples (Photo, in green) in two principal components. The first principal component (PC1) explains 33.3% (*Aloe arborescens*), 37.1% (*Aloe barbadensis*), and 38.1% (*Aloe chinensis*), respectively, of the total variance in the data, revealing that the mean profiles of the Dark group and Photo group are significantly different. The second principal component (PC2) explains an additional and not negligible 18.7%, 22.4%, and 19.1% variance in the data.

The analysis disclosed that the treatment with Aloe EVs and subsequent photoexposition altered the SK-MEL-5 metabolome; in fact, dark samples and photoexposed samples treated with the three types of Aloe EVs were clearly separated along the first principal component (PC1 along the *x*-axis in Figure 5), indicating a differential pattern of the metabolites found in photoexposed SK-MEL-5 cells compared to SK-MEL-5 cells treated with EVs but kept in the dark.

The metabolomic analysis was also conducted on untreated SK-MEL-5 cells kept in the dark (CTRL Dark) and photoexposed (CTRL Photo) in the same conditions as those treated with EVs from the three Aloe species. However, no significant alterations were found in the metabolome of untreated photoexposed cells relative to CTRL Dark (Figure S2), revealing that the effect found in photoexposed EV-treated SK-MEL-5 cells is expressly related to the photosensitization of the delivered Aloe EV cargo and is not merely due to light exposure.

**Figure 6:**
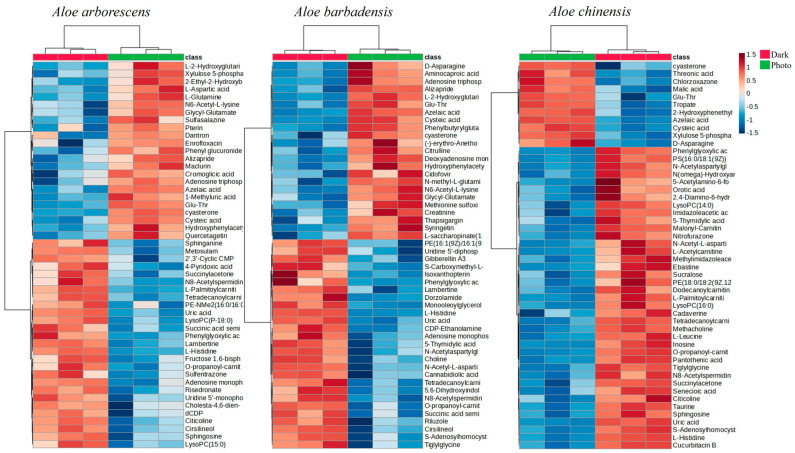
Heatmaps of SK-MEL-5 treated with *Aloe arborescens*, *Aloe barbadensis*, and *Aloe chinensis* EVs in the dark (red) and after photoexposition (green).

To further discriminate the Dark and Photo groups of treated cells, a statistical analysis was performed by using the t-test. In particular, in the case of photoexposed Aloe EV-treated cells, 68, 88, and 101 metabolites were annotated, respectively, representing 17%, 22%, and 25% of all of the detected metabolites (Tables S1–S3). A heatmap was then created to highlight the 50 significantly altered metabolites in the two metabolomes (Figure 6). The phenotypic expression of the fifty metabolites’ pathways was significantly altered after the treatment, revealing strong-separate clustering among the analyzed metabolomes. In particular, the rows show the metabolites, and the columns characterize the sample (red for the dark, green for the photoexposition). Significantly decreased metabolite regulation is shown in blue, while noticeably increased metabolites are presented in red. The alterations of these metabolites are mainly involved in the metabolism pathways of amino acid synthesis; in the metabolism of sphingolipids, glycerophospholipids, and fatty acids; and in the metabolism of purines and pyrimidines, indicating and confirming the evidence of strong cellular oxidative stress induced by the photoexposition and changes in cellular energy metabolism that could promote the apoptotic process.

In the case of untreated CTRL cells, the *t*-test (Table S4) identified only 20 metabolites in the CTRL Photo metabolome significantly different from those of CTRL Dark. Among these 20 metabolites, only 5 were also present in some of the metabolomes of photoexposed cells treated with EVs, though none of them are involved in oxidative stress pathways. This finding indicates that the major changes detected are specifically caused by the treatment of SK-MEL-5 cells with Aloe EVs and their subsequent photoexposition.

In particular, it can be seen how the irradiation after the treatment with the EVs of *Aloe arborescens* and *Aloe barbadensis* determines a similar clustering of the number of altered metabolites, with 29 and 27 metabolites decreased and 21 and 23 increased, respectively. The photoexposition with *Aloe chinensis* EVs instead induced a different clustering with 39 decreased and only 11 increased metabolites.

**Figure 7:**
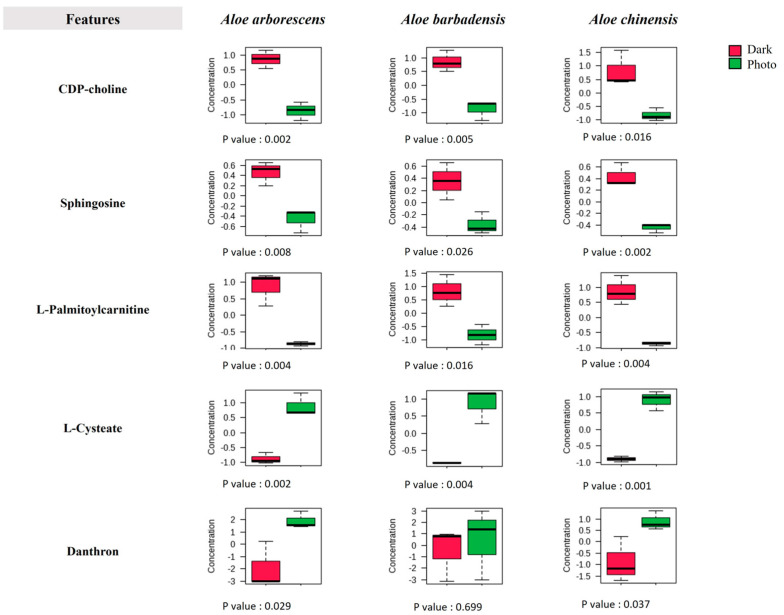
Representative metabolite levels in photoexposed SK-MEL-5 cells (green) and control cells kept in the dark (red). Values were evaluated from integrated isolated signals for each metabolite present in Q-TOF LC/MS spectra and scaled in MetaboAnalyst.

Some of the pathways that were significantly altered and potentially involved in the process of oxidative stress and possibly apoptosis induced by treatment with Aloe EVs and photoexposure were therefore analyzed. The measurements were carried out with MetaboAnalyst to identify some of the key molecules involved in the altered metabolic pathways. The results are shown as Box Plots in Figure 7.

A significant decrease in the metabolism of CDP-choline and choline was found following treatment with EVs from the three Aloe species and subsequent photoexposure. Both choline and CDP-choline are two key molecules in the metabolism of glycerophospholipids; therefore, their depletion following photoexposition could indicate strong oxidative stress damaging the main structural components of the plasma membranes. In fact, choline is a precursor of phosphatidylcholine (PC) and acetylcholine, also playing a fundamental role in signal transduction in the cell membrane [53]. Furthermore, the depletion in choline levels determines an altered composition of the mitochondrial membrane and an alteration of the β-oxidation of fatty acids. These conditions occur following an increase in ROS concentration and cellular apoptosis phenomena [54,55]. In a study conducted by Lu et al., it was demonstrated that the treatment of hepatocellular carcinoma cells with natrin determined a decrease in the CDP-choline level, blocking cell progression and inducing apoptosis [56]. Cancer is usually associated with an increase in phospholipid metabolism due to the high demand for membrane synthesis and intracellular signaling; therefore, a decrease in key molecules of sphingolipid metabolism, such as choline, indicates that cells show a positive response to treatment [57,58]. Even in the case of sphingosine, a key lipid in the metabolism of sphingolipids, a significant decrease was found following the irradiation of treated cells. Indeed, in many cancers, sphingolipid metabolism is usually upregulated with an increase in key metabolites. It is widely recognized that sphingosine 1-phosphate (S1P), deriving from sphingosine, plays a fundamental role in the growth and progression of cancer; therefore, the reduction level of sphingosine in samples treated with the three types of EVs and the subsequent photoexposition allows an actual functioning to be hypothesized for the tested system [55,56,58].

In all metabolomes, a decrease in the levels of L-palmitoylcarnitine, which has the task of facilitating the transport of long-chain fatty acids from the cytosol to the mitochondria for beta-oxidation, was also detected. Its decrease therefore leads to an accumulation of these long-chain fatty acids, which expose the cell to greater lipid oxidation and acetyl-CoA depletion, compromising the tricarboxylic acid (TCA) cycle. Usually, in the tumor microenvironment, there is an altered metabolism of fatty acids whose oxidation can provide energy to promote the growth of tumor cells; therefore, high levels of lipid markers such as L-palmitoylcarnitine are typically associated with a state of tumor progression. In this case, the treatment with EVs, especially from *Aloe arborescens* and *Aloe chinensis*, together with photoexposition, seems to have blocked this mechanism [59].

The amino acid metabolism was also deeply altered following the treatment; in fact, in all three metabolomes, the level of histidine, one of the essential amino acids for cell proliferation, significantly decreased, as well as the metabolism of glutamine and aspartic acid (*A. arborescens*), or asparagine (*A. barbadensis* and *A. chinensis*). Noteworthy is the increase in cysteic acid resulting from the irreversible oxidation of cysteine in all three metabolomes. The presence of this molecule supports the hypothesis of oxidative stress induced by the photoexposition as it has been demonstrated that cysteic acid is formed in an environment characterized by a high concentration of ROS. In this case, it thus represents an important marker of oxidative stress and protein deterioration [60,61].

Finally, an increase in the levels of danthron, an anthraquinone derivative with peculiar properties, was found in the metabolomes of the photo-treated cells. In particular, its increase was significant in the metabolomes of SK-MEL-5 cells treated with EVs from *Aloe arborescens* and *Aloe chinensis* with subsequent photoexposition. This compound represents the oxidative form of anthraquinone, formed by the replacement of two hydrogen atoms with hydroxyl groups, and its increase could indeed be related to the high presence of ROS in the cellular microenvironment following photoexposure. Danthron works by inducing DNA damage and depolarizing the mitochondrial membrane, which determines the activation of the apoptotic cascade. Previous studies have shown that this compound is strongly anti-carcinogenic, inducing DNA damage and apoptosis in both malignant stomach cancer and glioblastoma cells [62,63]. The increased level of danthron in the three metabolomes of photoexposed SK-MEL-5 cells treated with the EVs of the different Aloe species corroborates the hypothesis that Aloe EVs can serve as a carrier of photoactive compounds capable of inhibiting tumor growth and therefore be potentially valuable as photosensitizers in the photodynamic therapy of cancer.

## 4. Conclusions

In summary, the effects of EVs from three different Aloe species on human melanoma cells have been analyzed, evaluating their phototoxic effect attributable to their anthraquinone content. In fact, anthraquinones are considered natural photosensitizers capable of inducing ROS production in the presence of light and oxygen, triggering a cascade of events that cause oxidative stress and supposedly apoptosis. EVs are able to protect their labile cargo from degradation and allow its uptake by recipient cells; in this way, bioactive molecules such as anthraquinones can be easily internalized by the cell and carry out their function under light irradiation. The phototoxic effect of Aloe EVs was closely related to the production of ROS, which induced strong oxidative stress in melanoma cells, as corroborated by the metabolome analysis. Furthermore, comparing the phototoxic effects of plant extracts to those of EVs, it becomes clear that EVs, despite containing lower levels of photoactive anthraquinones, possess a higher efficacy in delivering them into cells by virtue of their natural ability to transfer cargos for cell-to-cell communication, consequently leading to an amplified effect compared to the original crude extract. This suggests that EVs from *Aloe arborescens*, *Aloe barbadensis*, and *Aloe chinensis*, albeit to different extents, offer a promising route for targeted delivery of photoactive compounds in a more efficient manner compared to traditional plant extracts, and may be used as potential photosensitizers in the photodynamic therapy of cancer.

## Data Availability

The original contributions presented in the study are included in the article/Supplementary Materials, further inquiries can be directed to the corresponding author.

## Supplementary Materials

The following supporting information can be downloaded at: https://www.mdpi.com/article/10.3390/cells13221845/s1, Figure S1: Cytotoxicity and phototoxicity of Aloe sp. EVs and extracts; Figure S2. PCA score plots of untreated SK-MEL-5 cells; Table S1: *t*-test results of photoexposed SK-MEL-5 cells treated with *Aloe a.* EVs; Table S2: *t*-test results of photoexposed SK-MEL-5 cells treated with *Aloe b.* EVs; Table S3: *t*-test results of photoexposed SK-MEL-5 cells treated with *Aloe c.* EVs; Table S4: *t*-test results of photoexposed untreated SK-MEL-5 cells.
