# Supplementary material for: Aloe Extracellular Vesicles as Carriers of Photoinducible Metabolites Exhibiting Cellular Phototoxicity
Source: Cells. 2024 Nov 7;13(22):1845. doi: 10.3390/cells13221845 (PMC11592872; doi:10.3390/cells13221845)
Supplement: Supplementary file 1 [file cells-13-01845-s001.zip › cells-3248977-supplementary.pdf]

Supplementary Material

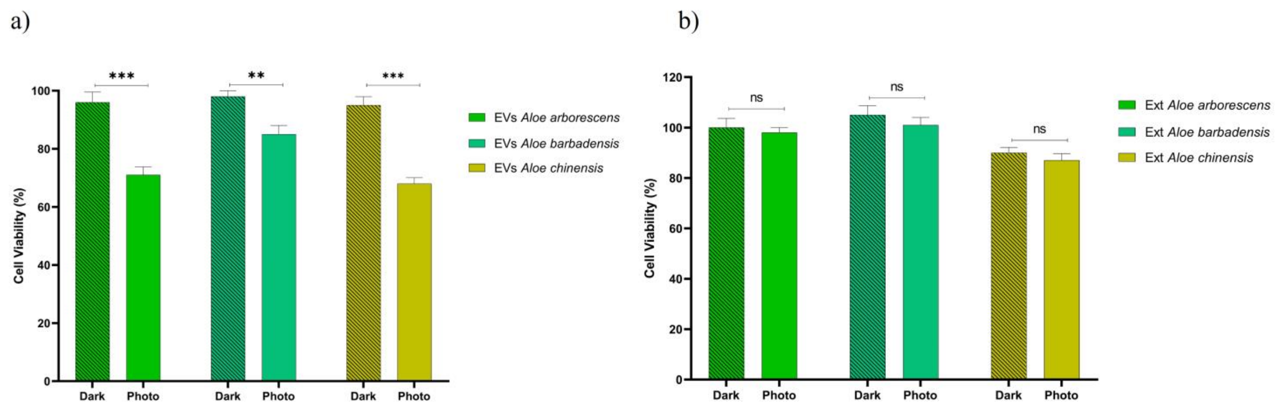

Figure S1. Cytotoxicity and phototoxicity of Aloe sp. EVs (a) and Extracts (Ext) (b), both diluted in a 1:100 ratio, on SK-MEL-5 cells. Cytotoxicity has been evaluated on cells kept in the dark (Dark), while phototoxicity has been assessed after 50 min of irradiation (corresponding to irradiation energy of 2.55 J/cm^2, λ_{exc} = 390−400 nm) (Photo). Cell viability is expressed as the mean of three independent experiments of four replicas each ±SD; 100% corresponds to control (untreated cells kept in the dark and photoexposed for Dark and Photo, respectively) mean values. $^{**}p < 0.01$, $^{***}p < 0.001$.

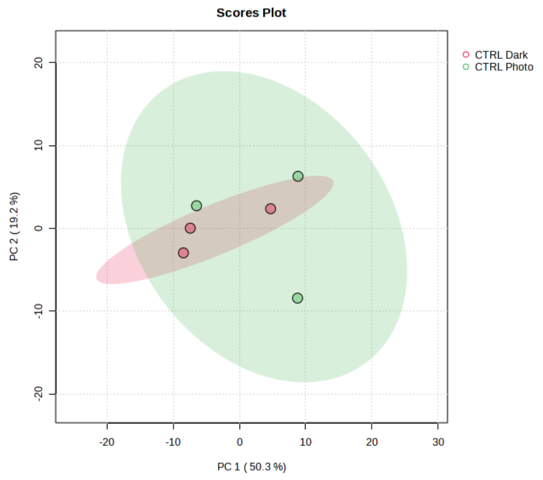

Figure S2. PCA score plots of untreated SK-MEL-5 cells kept in the dark (CTRL Dark, red) and after photoexposition (CTRL Photo, green).

Table S1: t-test results of photoexposed SK-MEL-5 cells treated with *Aloe a.* EVs for metabolomic analysis.

Photoexposed Aloe arborescens EV-treated SK-MEL-5 cells	**t.stat**	**FDR**
Cyasterone	-20.887	0.01299
L-Histidine	17.771	0.01299
Glu-Thr	-15.049	0.0167
Uric acid	9.4263	0.072989
Lambertine	9.0458	0.072989
L-Palmitoylcarnitine	8.5328	0.076088
Azelaic acid	-7.5415	0.10296

DG(18:1(9Z)/18:1(9Z)/0:0)	7.1398	0.10296
1-Methyluric acid	-7.0796	0.10296
LysoPC(P-18:0)	6.2528	0.14469
Phenylglyoxylic acid	6.095	0.14469
Cysteic acid	-5.8921	0.14469
Tetradecanoylcarnitine	5.8478	0.14469
Metosulam	5.5282	0.16477
Hydroxyphenylacetylglycine	-5.0191	0.21566
Citicoline	4.9175	0.21566
Quercetagetin	-4.8543	0.21566
Maclurin	-4.5878	0.22941
Succinic acid semialdehyde	4.5489	0.22941
Sphinganine	4.5183	0.22941
Cirsilineol	4.4441	0.22941
L-Aspartic acid	-4.418	0.22941
Risedronate	4.3703	0.22941
Adenosine monophosphate	4.3006	0.23225
L-Glutamine	-4.1657	0.2392
L-2-Hydroxyglutaric acid	-4.1637	0.2392
Cilostazol	4.0809	0.24154
N8-Acetylspermidine	4.061	0.24154
Phenyl glucuronide	-3.932	0.25291
Adenine	-3.9229	0.25291
2-Ethyl-2-Hydroxybutyric acid	-3.8499	0.25505
LysoPC(15:0)	3.8283	0.25505
Sulfasalazine	-3.8011	0.25505
Uridine 5'-monophosphate	3.7484	0.25909
Uridine 5'-diphosphate	3.6793	0.26733
Sphingosine	3.5489	0.26788
O-propanoyl-carnitine	3.5083	0.26788
Fructose 1,6-bisphosphate	3.4888	0.26788
Methionine sulfoxide	-3.4797	0.26788
Sulfentrazone	3.4556	0.26788
Succinylacetone	3.4122	0.26788
Xylulose 5-phosphate	-3.4046	0.26788
Liquiritin	3.3935	0.26788
2',3'-Cyclic CMP	3.3803	0.26788
PE-NMe2(16:0/16:0)	3.3794	0.26788
Glutaminylglycine	-3.3598	0.26788
4-Pyridoxic acid	3.3507	0.26788
Glycyl-Glutamate	-3.2832	0.27939
2-Hydroxyphenethylamine	-3.2506	0.28223
N6-Acetyl-L-lysine	-3.1852	0.28753
Adenosine triphosphate	-3.1561	0.28753
L-Asparagine	-3.1544	0.28753
Alizapride	-3.1487	0.28753
Sulfamethoxazole	-3.1289	0.28764

(2E)-3-(3,4-dihydroxyphenyl)-1-(2,4,6-trihydroxyphenyl)prop-2-en-1-one	-3.0312	0.30589
Mauritianin	-3.0284	0.30589
IDP	-2.9887	0.3125
Nicotinamide riboside	2.9624	0.3152
Thiamine	2.9249	0.31638
4-Hydroxyproline	-2.9204	0.31638
Pterin	-2.9058	0.31638
9(S)-HPOT	2.8711	0.31638
S-Adenosylhomocysteine	2.8689	0.31638
Cromoglicic acid	-2.8583	0.31638
L-3-Cyanoalanine	2.8328	0.31638
Silychristin	2.817	0.31638
dCDP	2.8151	0.31638
Chlorzoxazone	-2.7876	0.32057

Table S2: t-test results of photoexposed SK-MEL-5 cells treated with *Aloe b.* EVs for metabolomic analysis.

Photoexposed Aloe barbadensis EV-treated SK-MEL-5 cells	**t.stat**	**FDR**
L-Histidine	41.621	0.000878
Azelaic acid	-18.193	0.011838
L-2-Hydroxyglutaric acid	-15.111	0.016433
Monooleoylglycerol	11.202	0.034823
Cysteic acid	-10.827	0.034823
Uric acid	10.45	0.034823
Phenylbutyrylglutamine	-10.029	0.035017
Deoxyadenosine monophosphate	-7.9314	0.070828
Alizapride	-7.8038	0.070828
Dorzolamide	7.6027	0.070828
CDP-Ethanolamine	7.321	0.074257
Glu-Thr	-6.9933	0.079506
Syringetin	-6.8761	0.079506
N6-Acetyl-L-lysine	-6.6789	0.082296
Aminocaproic acid	-6.5037	0.084785
Adenosine triphosphate	-6.3778	0.085459
Tetradecanoylcarnitine	6.0781	0.094627
N-methyl-L-glutamic Acid	-5.9402	0.094627
Gibberellin A3	5.6688	0.094627
N-Acetyl-L-aspartic acid	5.6653	0.094627
5,6-Dihydroxyindole-2-carboxylic acid	5.5519	0.094627
S-Carboxymethyl-L-cysteine	5.55	0.094627
Lambertine	5.4831	0.094627
Cirsilineol	5.4544	0.094627
N8-Acetylspermidine	5.4432	0.094627
Citrulline	-5.4303	0.094627
L-saccharopinate(1-)	-5.2666	0.098188
PE(16:1(9Z)/16:1(9Z))	5.2141	0.098188

Isoxanthopterin	5.2133	0.098188
D-Asparagine	-5.0412	0.10477
Adenosine monophosphate	4.9895	0.10477
Hydroxyphenylacetylglycine	-4.9164	0.10477
Choline	4.905	0.10477
5-Thymidylic acid	4.8942	0.10477
Glycyl-Glutamate	-4.7913	0.1076
Methionine sulfoxide	-4.7503	0.1076
O-propanoyl-carnitine	4.7367	0.1076
Creatinine	-4.7054	0.1076
N-Acetylaspartylglutamic acid	4.6171	0.11197
Cannabidiolic acid	4.4956	0.11972
Phenylglyoxylic acid	4.4051	0.12451
Cyasterone	-4.3819	0.12451
S-Adenosylhomocysteine	4.287	0.13103
Succinic acid semialdehyde	4.1476	0.14082
Cidofovir	-4.1301	0.14082
Riluzole	4.0777	0.14082
Thapsigargin	-4.0683	0.14082
Tiglylglycine	4.0307	0.14082
Uridine 5'-diphosphate	4.0177	0.14082
(-)-erythro-Anethole glycol 2-glucoside	-4.0123	0.14082
LysoPE(18:1(9Z)/0:0)	3.7838	0.16633
Citicoline	3.7695	0.16633
L-Glutamine	-3.7077	0.1722
Sphingosine	3.6552	0.17699
gamma-Glutamylalanine	-3.5397	0.18727
Sulfentrazone	3.5369	0.18727
2-Hydroxyphenethylamine	-3.5312	0.18727
Entecavir	3.5085	0.18785
Phthalic acid	-3.4349	0.19354
L-Aspartic acid	-3.4233	0.19354
4-Guanidinobutanoic acid	-3.4075	0.19354
L-Palmitoylcarnitine	3.4027	0.19354
2,5-Furandicarboxylic acid	-3.3814	0.19425
L-Methionine	-3.2988	0.20648
6-Methylthiopurine	3.2792	0.20708
Uridine 5'-monophosphate	3.2274	0.21133
Gluconic acid	-3.2002	0.21133
PC(16:0/18:2(9Z,12Z))	3.199	0.21133
Xylulose 5-phosphate	-3.1948	0.21133
Imidazoleacetic acid	3.1646	0.21246
Orotic acid	3.1506	0.21246
L-Arginine	3.1448	0.21246
Taurolithocholic acid 3-sulfate	3.1121	0.21281
PC(22:6(4Z,7Z,10Z,13Z,16Z,19Z)/22:6(4Z,7Z,10Z,13Z,16Z,19Z))	-3.1024	0.21281
Inosine	3.1008	0.21281

Tropate	-3.0666	0.21711
PE-NMe(18:1(9Z)/18:1(9Z))	3.0477	0.21828
LysoPC(15:0)	3.0319	0.21885
Phenyl glucuronide	-3.018	0.21905
Formononetin	-2.9796	0.22467
Quercetagetin	-2.9537	0.22767
5-Acetylamino-6-formylamino-3-methyluracil	2.9148	0.23378
1-Naphthol	2.9025	0.23381
Aminoadipic acid	2.8817	0.23563
N-a-Acetyl-L-arginine	-2.8674	0.23563
9-OxoODE	2.8592	0.23563
Asymmetric dimethylarginine	2.8399	0.23563
Felodipine	-2.8368	0.23563

Table S3: t-test results of photoexposed SK-MEL-5 cells treated with *Aloe c.* EVs for metabolomic analysis.

Photoexposed Aloe chinensis EV-treated SK-MEL-5 cells	**t.stat**	**FDR**
Uric acid	25.341	0.00635
O-propanoyl-carnitine	18.057	0.010141
L-Histidine	15.883	0.010141
Azelaic acid	-15.282	0.010141
Phenylglyoxylic acid	14.555	0.010141
PS(16:0/18:1(9Z))	12.74	0.010141
Tetradecanoylcarnitine	12.562	0.010141
Cysteic acid	-12.401	0.010141
Methacholine	12.22	0.010141
Pantothenic acid	12.108	0.010141
Cucurbitacin B	12.101	0.010141
S-Adenosylhomocysteine	12.005	0.010141
Inosine	11.597	0.010715
L-Leucine	11.192	0.01143
Tiglylglycine	10.969	0.011539
Taurine	10.603	0.012154
Malonyl-Carnitin	10.456	0.012154
N-Acetylaspartylglutamic acid	10.327	0.012154
L-Palmitoylcarnitine	9.6082	0.015224
Xylulose 5-phosphate	-9.2102	0.017025
Threonic acid	-8.6188	0.02023
Chlorzoxazone	-8.5897	0.02023
LysoPC(16:0)	8.2434	0.022006
N(omega)-Hydroxyarginine	8.2134	0.022006
Sphingosine	7.9262	0.024191
Nitrofurazone	7.8082	0.024624
D-Asparagine	-7.2845	0.030828
N8-Acetylspermidine	7.195	0.031142
Dodecanoylcarnitine	6.9699	0.033878
N-Acetyl-L-aspartic acid	6.9064	0.03389

Tropate	-6.8233	0.034316
Succinylacetone	6.5761	0.036629
L-Acetylcarnitine	6.5643	0.036629
LysoPC(14:0)	6.5406	0.036629
2-Hydroxyphenethylamine	-6.3422	0.039884
Imidazoleacetic acid	5.6857	0.057235
Senecioic acid	5.6506	0.057235
Sucralose	5.6188	0.057235
Malic acid	-5.5513	0.058261
Methylimidazoleacetic acid	5.4306	0.061495
Cyasterone	-5.1735	0.071378
Citicoline	5.0655	0.074654
Cadaverine	5.0406	0.074654
Glu-Thr	-4.9974	0.075211
Ebastine	4.952	0.075954
Orotic acid	4.8291	0.07676
5-Acetylamino-6-formylamino-3-methyluracil	4.8107	0.07676
PE(18:0/18:2(9Z,12Z))	4.8039	0.07676
5-Thymidylic acid	4.7976	0.07676
2,4-Diamino-6-hydroxypyrimidine	4.7913	0.07676
5,6-Dihydroxyindole-2-carboxylic acid	4.633	0.084614
Cilostazol	-4.5518	0.08687
Phenyl glucuronide	-4.5362	0.08687
Lambertine	4.5226	0.08687
N-Acetylmethionine	4.4871	0.087641
(R)-(E)-Sulforaphene	-4.428	0.090085
Oxidized glutathione	-4.3231	0.09581
LysoPC(15:0)	4.3045	0.09581
Acetylglycine	4.1936	0.10209
Iminodiacetic acid	4.1826	0.10209
2-Amino-3-hydroxypropanoic acid	3.9594	0.11737
Tetraethylene glycol	-3.9589	0.11737
D-Tryptophan	-3.9496	0.11737
Uridine 5'-monophosphate	3.9348	0.11737
LysoPE(18:0/0:0)	3.8924	0.11928
PE(16:1(9Z)/16:1(9Z))	3.8793	0.11928
PE(16:0/18:2(9Z,12Z))	3.82	0.12282
Pyrrole-2-carboxylic acid	3.8065	0.12282
PE(16:0/18:1(9Z))	3.7932	0.12282
L-Glutamine	-3.77	0.12351
Betaine	3.7078	0.12853
LysoPC(18:0)	3.6747	0.13048
PC(18:1(9Z)/14:0)	3.6	0.13602
Choline	3.5968	0.13602
LysoPE(18:1(9Z)/0:0)	3.5136	0.14235
Scopoletin	-3.513	0.14235
Glycine	-3.5019	0.14235

Glucose isomerase from streptomyces rubiginosus	-3.3971	0.15384
9-OxoODE	3.374	0.15384
N6-(delta2-Isopentenyl)-adenine	-3.3721	0.15384
6-Methylthiopurine	3.3619	0.15384
(+)-Zeylenol	3.3248	0.15729
5-(2-Hydroxyethyl)-4-methylthiazole	3.2926	0.15972
Gamma-Aminobutyric acid	3.2781	0.15972
Sulfentrazone	3.2702	0.15972
2,3-Dihydroxy-3-methylbutanoate	3.1978	0.16907
PE-NMe2(16:0/16:0)	3.1477	0.17151
SM(d18:1/24:1(15Z))	3.1473	0.17151
Adenosine monophosphate	3.147	0.17151
4-Chloro-5-sulfamoylanthranilic acid	3.119	0.17425
Rhaponticin	3.0298	0.18797
L-Aspartic acid	-2.9946	0.19248
Dantron	-2.9774	0.19287
repaglinide aromatic amine	2.9617	0.19287
Riluzole	-2.9601	0.19287
Cyclopentolate	2.9252	0.19762
Adenosine triphosphate	-2.8906	0.20248
Trichlormethiazide	-2.8472	0.20938
LysoPC(17:0)	2.8166	0.21379
Diethylcarbamazine	-2.7942	0.21542
CDP-Ethanolamine	2.7813	0.21542

Table S4: t-test results of photoexposed untreated SK-MEL-5 cells (CTRL Photo) for metabolomic analysis.

SK-MEL-5 CTRL Photo	**t.stat**	**FDR**
Galactonic acid	-9.7566	0.27264
Quinestrol	-5.8828	0.58827
Quercetagetin	5.5619	0.58827
Tropate	5.4978	0.58827
5-Hydroxymethyl tolterodine	-4.603	0.82278
LysoPC(17:0)	-4.4561	0.82278
Talinolol	-4.0497	0.87477
LysoPC(P-18:0)	-3.5606	0.87477
Valdecoxib	3.5189	0.87477
repaglinide aromatic amine	3.4669	0.87477
Indolelactic acid	3.4331	0.87477
Sumiki's acid	3.3813	0.87477
Daidzein	3.2632	0.87477
Oleamide	-3.262	0.87477
Cefoperazone sodium	3.0191	0.87477
Dorzolamide	-2.9643	0.87477
Propranolol	2.9527	0.87477
Clenbuterol	-2.8952	0.87477
CDP	-2.8837	0.87477
3-Sulfino-alanine	-2.8317	0.87477
